# A Remote-Controlled Robotic System with Safety Protection Strategy Based on Force-Sensing and Bending Feedback for Transcatheter Arterial Chemoembolization

**DOI:** 10.3390/mi11090805

**Published:** 2020-08-25

**Authors:** Junqiang Zhou, Ziyang Mei, Jia Miao, Jingsong Mao, Lingyun Wang, Dezhi Wu, Daoheng Sun, Yang Zhao

**Affiliations:** 1 Department of Mechanical and Electrical Engineering, Xiamen University, Xiamen 361102, China; zhoumark95@stu.xmu.edu.cn (J.Z.); umeko@stu.xmu.edu.cn (Z.M.); miaojia@stu.xmu.edu.cn (J.M.); wangly@xmu.edu.cn (L.W.); wdz@xmu.edu.cn (D.W.); sundh@xmu.edu.cn (D.S.); 2 Department of Radiology, Xiang’an Hospital of Xiamen University, Xiamen 361102, China; maojingsong@xmu.edu.cn

**Keywords:** interventional robot, master–slave robotic system, force-sensing, bending feedback, motion constraint framework

## Abstract

Transcatheter arterial chemoembolization (TACE) is the common choice of non-open surgery for hepatocellular carcinoma (HCC) now. In this study, a simple TACE robotic system of 4-degree-of-freedom is proposed to get higher accuracy and stability of the surgery operation and reduce X-ray exposure time of the surgeons. The master–slave control strategy is adopted in the robotic system and a customized sigmoid function is designed to optimize the joystick control of the master–slave robotic control system. A force-sensing module is developed to sense the resistance of the guide wire in linear delivery motion and an auxiliary bending feedback method based on constraint pipe with a film sensor is proposed. With two force-sensing methods, the safety strategy of robotic motion with 9 different motion constraint coefficients is given and a human–computer interface is developed. The TACE robot would monitor the value of the force sensor and the analog voltage of the film sensor to adopt the corresponding motion constraint coefficient in every 10 ms. Vascular model experiments were performed to validate the robotic system, and the results showed that the safety strategy could improve the reliability of the operation with immediate speed constraint and avoid potential aggressive delivery.

## 1. Introduction

Vascular interventional surgery (VIS) is widely used in treating tumors because of its trauma with minimal hemorrhage and short recovery time. As it is a common regional chemotherapy, transcatheter arterial chemoembolization (TACE) belongs to VIS which infuses anticancer drugs or embolic agents into hepatic artery through femoral artery intubation. Its curative effects have been extensively confirmed and it is the common choice of non-open surgery for hepatocellular carcinoma (HCC) [1,2]. Compared with other interventional surgeries, liver interventional surgery is relatively simple. However, it also needs to perform the surgery under the guidance of digital subtraction angiography (DSA) system for hours to position the suitable type guide wire and catheter and at the planned location.

The experience of the surgeons in interventional surgery greatly affects the quality of operations, but long period of radiation exposure does cause great harm to surgeons’ health [3]. Trace back to 2005, a computerized interventional remote navigation system (Navi Cath, Israel) was reported, the said system assisted the placement of stents in 18 patients successfully [4], and during the surgery, surgeons avoided the high doses of X-ray radiation and did not need to wear heavy lead suit. After that, researchers have been increasingly interested in introducing teleoperated interventional robotic system into the relevant surgery operations. A reliable robotic surgical system could help surgeons control the guide wire/catheter to achieve the same surgical results remotely [5]. The collaboration of master–slave controlled system could help to reduce X-ray radiation on surgeons that often require the performance of several operations a day. In addition, combined with reasonable signals capture mechanism, robotic system could realize feedback response in a smaller time scale before surgeons react [6].

Compared with surgical robots such as Da Vinci^®^ [7,8], which need appropriate modeling of the interaction between the manipulated tissues and the instruments, the interventional surgical robot is relatively simple and are mainly developed to realize the delivery and rotation of the guide wire/catheter in the blood vessel. Reliability and ease of use are the core competitiveness of medical robots. Research on interventional surgery robots has been carried out by institutes and universities around the world. The previous work was mainly focused on how to achieve the transformation of master–slave movement with reasonable mechanical structures. Guo designed a friction-wheel driving mechanism and the surgery experiment was carried out successfully in phantom model, micro force sensor and fiber optic pressure sensor were proposed to be fixed at the tip of the catheter to evaluate delivery situation [9,10]. Wang designed a similar friction-wheel driving mechanism and the remote-controlled delivery and rotation of the guide wire was performed successfully to an adult dog in their robotic surgery, they also tried to wrap the tip of the guide wire with polyvinylidene fluoride (PVDF) to detect the contact force of the delivery operation [11,12]. In addition to design friction-wheel robot structure, Fu also tried to make the catheter itself into a robot system with shape memory alloy (SMA) material and adjust the shape of its tip by adjusting the current [13].

With the development of interventional robots, reliable robot structural design remains a key concern, meanwhile, the safety, simplicity, and reliability of interventional robot system become increasingly important in relevant system design. Yang proposed a twirling finger structure to imitate the surgeons twirling motion of the guide wire and they used a lever structure to measure the resistance of delivery motion of the guide wire [14]. Cha designed an ingenious assembly-type robot which combined a friction wheel and a telescopic rod structure while the change of the motor current was used to assess the resistance force during the delivery movement of the guide wire [15,16]. Shen developed a robot system based on electric slider and gear mechanism module for cerebrovascular surgery and an eccentricity spring control model was proposed to reduce the hand jitter captured by the joystick [17]. Feng tried to change the motion scaling factor between the master side and the slave side according to the distal end position, the reliability of delivery and rotation movements were improved [18]. Zhao converted the surgeons’ experience into numerical strategies about the force feedback of fusion rules to ensure the safety of relevant robot in vascular interventional procedures [19]. In addition, Guo optimized the structure of the robot and developed master-salve algorithm in recent years, they attempted to capture the delivery resistance with a slider clamping structure [20,21,22,23,24].

In the past few years, some remote-controlled interventional vascular surgery robots have also been developed by commercial companies. Specifically, the interventional surgical robot system mainly includes those of Sensei^®^ Robotic System (Hansen Medical Inc., Mountain View, CA, USA) [25,26], Amigo^®^ Robot System (Catheter Precision Inc., Ledgewood, NJ, USA) [27] and CorPath^®^ Robot System (Corindus Robotics Inc., Waltham, MA, USA) [28]. Slippage of the friction-wheel or friction-belt driving method are mainly adopted in these commercial robots. The guide wire (0.18 or 0.35 mm diameter often, with a smooth surface) is clamped by the squeeze of the wheel or belt, relative slippage displacement of the guide wire and the clamping point is needed in the delivery movement and it would affect the delivery accuracy. Another problem is that the friction-wheel or friction-belt driving is difficult to realize the detection of the guide wire delivery resistance with relevant suitable structural, the methods of detecting the delivery resistance are not developed in these introduced commercial robots.

Based on the above literature illustrated, the future development trends of the interventional robots should be light structure, accurate force-sensing, and reasonable master–slave motion control. Although the force-measuring mechanism of the interventional robot system presented is often designed bulky, a few interventional robots could realize the cooperative movements of the catheter and the guide wire or make auxiliary judgments proactively to ensure the safety of the operation. In this paper, a remote-controlled robotic system is designed for TACE liver interventional surgery, a light force-sensing module and an auxiliary bending feedback force-measuring method are discussed.

## 2. The TACE Robotic System

**Figure 1:**
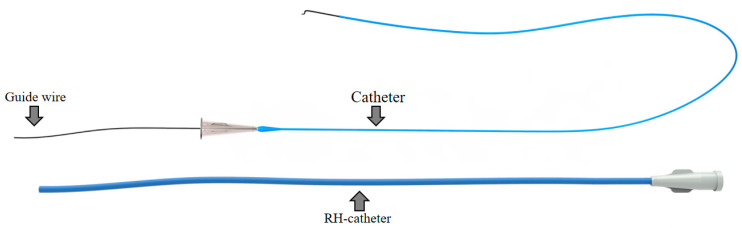
Schematic diagram of typical TACE tools.

The interventional surgical instrument used in a typical TACE for HCC is shown in Figure 1, the specification of the interventional instrument is chosen according to the condition of the patient.

**Figure 2:**
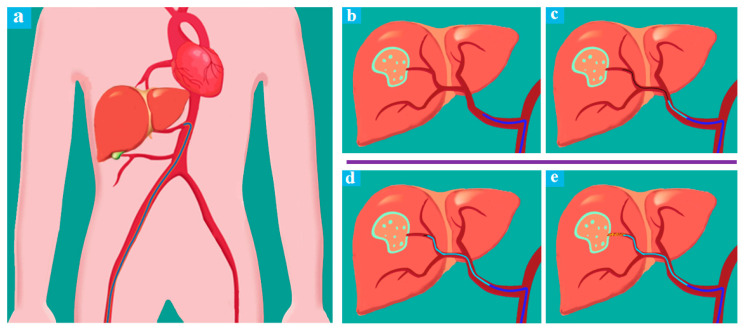
Conventional TACE process. (**a**) RH catheter insertion; (**b**) RH catheter placement; (**c**) Guide wire placement; (**d**) Catheter placement; (**e**) Guide wire removed and embolism injection.

Figure 2 illustrates a typical conventional procedure of liver interventional surgery. With the aid of DSA, the location of one liver tumor of the patient could be determined preliminarily. First, surgeons perform arterial puncture and place the right hepatic (RH) angiographic catheter (Figure 2b), the surgeons insert the guide wire and the catheter close to the target artery through the RH catheter (Figure 2c), above mentioned completed, the guide wire would be removed and surgeons inject contrast media through the catheter to confirm the target position, then reinsert the guide wire. After that, the surgeon will twist and advance the guide wire and the catheter repeatedly until they are sure to be placed at the right position (Figure 2d), and this period would take most of the operation time and cause a high dose of continuous radiation to the surgeons. Finally, the guide wire will be removed again, and embolic agents or other therapeutic agents would be injected to the target valley (Figure 2e). During a conventional TACE operation for HCC, X-rays are used inevitably, the TACE robot is designed to perform the remote-controlled operation of the guide wire/catheter and realize the function of Figure 2c,d, surgeons could work with the aid of DSA outside the operating room to perform TACE robotic assistant surgery.

### 2.1. Mechanical Structure

**Figure 3:**
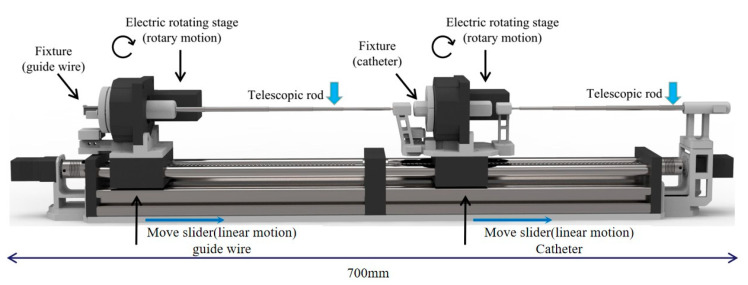
The mechanical structure of the TACE robot.

The mechanical of the TACE robot is shown in Figure 3. The TACE robot system has 4 DOFs includes a delivery motion and a rotary motion for the catheter and the guide wire. In the design, two lead screw linear moving platforms (8 mm thread lead) are adopted to realize the linear motion. The guide wire and the catheter are clamped by the fixture on the linear moving platforms. The motor of lead screw is from Pk513pa-h50s (Shanghai Oriental Motor Co., Ltd., Shanghai, China) and the overall length of the robot is 700 mm.

The rotary motion of the guide wire/catheter in the TACE robot is realized by an electrical servo rotation stage (Dg60-Asak, Shanghai Oriental Motor Co., Ltd.). The modules of the embedded gear in the rotation stage is 0.3. It could achieve better control stability and more transmission ratio accuracy. Meanwhile, to prevent the guide wire and the catheter from bending in delivery, two telescopic rods are adopted to assist the linear delivery movement. Table 1 shows the movement parameters of the robot and it could meet the needs of the TACE operation according to the surgeons’ evaluations.

**Figure 4:**
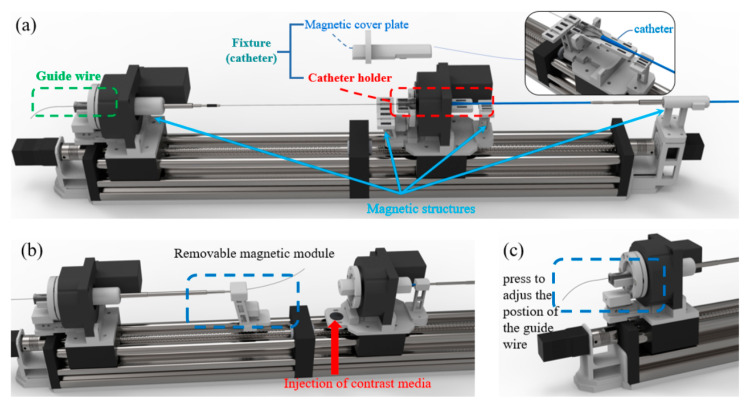
Mechanical structure details (**a**) Preoperative preparation of the catheter and the guide wire; (**b**) Removable magnetic module; (**c**) Position adjustment of the guide wire.

The working details of the TACE robot is shown in Figure 4. As shown in Figure 4a, the guide wire is clamped by the designed adjustable fixture, and the end of the catheter’s sheath is placed in the catheter holder which could be covered by the corresponding magnetic cover plate. Figure 4b shows the simple design of contrast medium injection, the fixing part of the telescopic rod is adsorbed in the movable slider by magnetic. It can be removed if necessary and the contrast media could be injected from the back without removing the guide wire completely by the surgeon. In addition, as shown in Figure 4c, before the robotic surgery begins, the surgeon could adjust the position of the guide wire expediently by pressing the adjustable fixture to place the guide wire at the established position to reduce the operation time.

### 2.2. Remote-Controlled System

**Figure 5:**
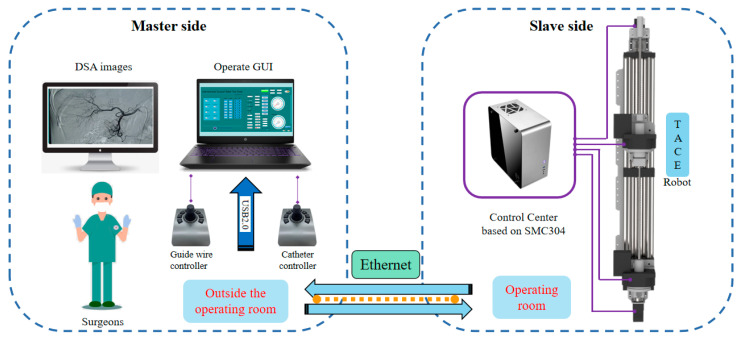
The schematic of the TACE robot remote-controlled system.

The schematic of the TACE robot remote-controlled system is shown in Figure 5, the TACE robot is assumed to cooperate with DSA display images to perform surgery in an actual robotic surgery scene. The system is composed of two master joysticks, a slave robot manipulator, a control box, and a computer. The control system is built on SMC304 programmable motion controller (Shenzhen Leadshine Control Technology Co., Ltd., Shenzhen, China). The response time of the slave robot is within 10 milliseconds based on Ethernet. Speed control mode based on joystick and displacement control mode based on encoder are developed, the latter is not introduced in this paper.

Two joysticks (Ltd-SMC71-USB, Shenzhen Xiaolong Electric Co., Ltd., Shenzhen, China) are used to operate the guide wire and the catheter in the slave side based on USB communication, respectively. The handle rocker of the joystick is designed with an active spring to stay stationary when robot is not working.

**Figure 6:**
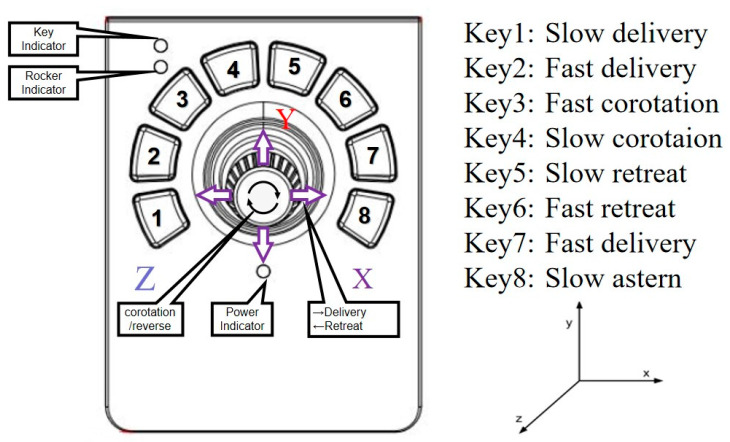
Functions of the joystick.

In speed control mode, the functions of the keys are defined in Figure 6. The signal output of the joysticks is adopted as the input of the movement speed of the linear delivery and rotation of the slave robot. Take the control of the guide wire as an example, the swing of the joystick can be transformed into a series of linear change of data value (*J_G)_*, it includes a group of X-direction data (*J_Gx_*) and a group of Z direction data (*J_Gz_*). The operation of the joystick by surgeon in X-direction is matched to the delivery speed and the direction of the guide wire (*V_Gx_*), while the rotation around Z axis is matched to the rotation speed and direction of the guide wire (*V_Gz_*). (The movement of Y axis is not linked)

Considering the trembling of hands would cause the fluctuation of joystick data, a simple and effective mapping algorithm between *V_Gx_* and *J_Gx_* is shown as follows:

(1) $$V_{Gx}=\left\{ \begin{array}{ll} \frac{1}{1+e^{-f(J_{Gx})+6}}V_{s-\max}Sgn(J_{Gx}),f(J_{Gx})=\frac{\left|J_{Gx}\right|}{20000}\times 25 & \left(|J_{Gx}|\leq 3000\right)\\ \frac{7J_{Gx}}{220000}V_{s-\max}+0.005V_{s-\max} & \left(3000<|J_{Gx}|\leq 25000\right)\\ \frac{1}{1+e^{-f(J_{Gx})+5.3}}V_{s-\max}Sgn(J_{Gx}),f(J_{Gx})=\frac{\left|J_{Gx}\right|}{30000}\times 8 & \left(25000<|J_{Gx}|\right) \end{array}\right.$$

**Figure 7:**
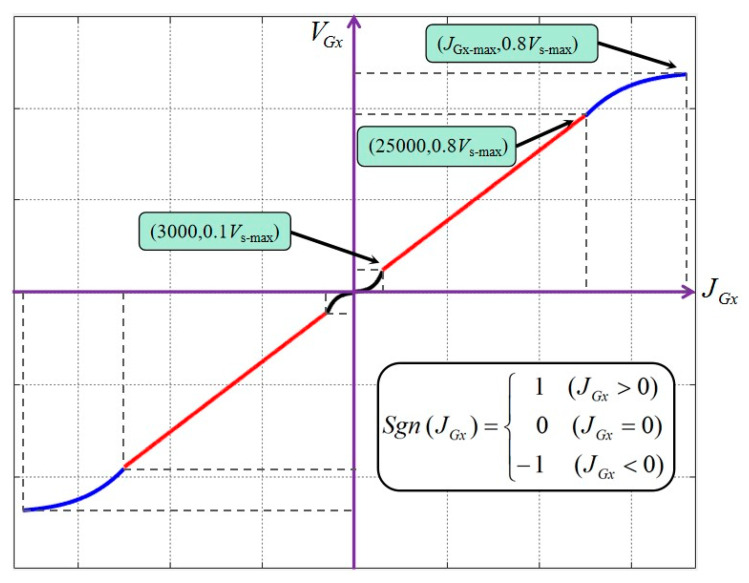
*J_Gx_*-*V_Gx_* data mapping.

The *J_G__x_*-*V_G*x*_* data mapping is shown in Figure 7. The positive and negative value of *V_Gx_* represent the forward and backward of the guide wire, Sgn (JGx) is a step function, and the Vs-max means the maximum axis speed which can be adjusted depending on the practical need.

Although the TACE robot works at low speed, the surgeon’s hand jitter would inevitably affect the operation accuracy, so the speed increase is designed to be smooth at the stage of low speed. In the stage of high speed, the tracking speed of the robot will be limited for the safety of the operation. At the same time, the change of the speed in the middle segment should be relatively sensitive that could meet the actual needs of the operation and speed up the operation. The surgeon can also select the key1 to key8 to realize the fine tune of the slave TACE robot position.

## 3. Force-Sensing and Safety Protection

In interventional surgery for HCC, the guide wire/catheter is operated to the target position with the aid of DSA by surgeons. During the conventional process of the surgery, the guide wire and catheter would produce haptic feedback for the decision of next delivery and rotation operation to surgeons from the delivery resistance, but the robot does not have the same perception as humans to prevent damage from the resistance or the collision. Therefore, a reliable force-sensing module for detecting the guide wire delivery resistance and an early auxiliary warning mechanism for evaluating catheter delivery condition level are proposed in the TACE robot.

### 3.1. Force-Sensing Module

The guide wire used for HCC interventional surgery is a J-shaped head guide wire, whose tip is very soft that can be molded into the desired shape. Even in actual surgery, the possible tip collision is difficult to be captured by hand, and it mainly depends on experienced surgeons’ judge of DSA images. Delivery speed of the surgeons is adjusted mainly by sensing the delivery resistance, therefore, a force-sensing module is designed in TACE robot for the detection of the guide wire delivery resistance. The delivery resistance of the guide wire could be displayed on computer in real time as a reference for the surgeons. In relevant interventional surgery model experiments, the resistance value is generally not greater than 2 N [29,30].

**Figure 8:**
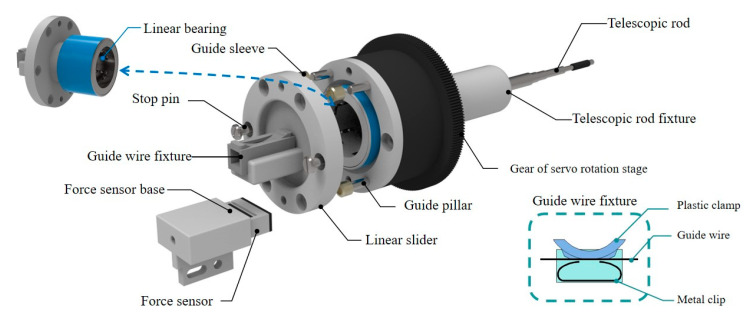
Components of the force-sensing module.

As shown in Figure 8, the fixture of the guide wire and the linear slider are fixed together. The resistance of the guide wire could be detected in the process of delivery movement by this structure.

The guide wire can be effectively clamped by pressing the plastic clamp above the metal clip, and the force sensor (FS09-5, INELTA Co., Ltd., Munich, Germany) will be pressed with the structure of liner slider and linear bearing when the guide wire encounters the resistance.

**Figure 9:**
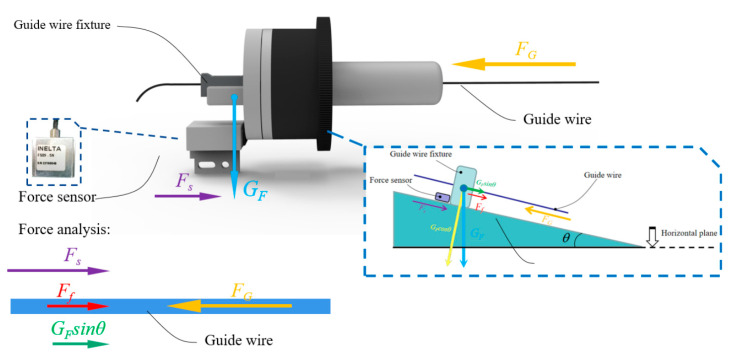
Principle for force analysis of the guide wire.

The principle of force analysis of the structure is shown in Figure 9. The resistance force of the delivery movement of the guide wire could be briefly summarized as follows:

(2) $$F_{G}-F_{f}-G_{F}\sin\theta =F_{s},F_{f}=\mu G_{F}\cos\theta$$

where *F_G_* is the resistance force of the guide wire advancement, *F_f_* is the friction force generated by the slight movement of the linear slider, the sleeve, the pillar, and the linear bearing. *G_F_* is the gravity of the guide wire fixture, *F_s_* is the detected force by the force sensor, *θ* is the angle between the robot manipulator arm and the horizontal, and *μ* is the coefficient of the friction between the slider and the linear bearing.

**Figure 10:**
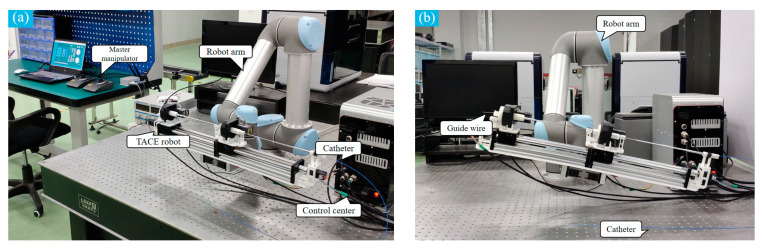
Diagram of the complete system of the TACE robot (**a**) Horizontal working state; (**b**) Inclined working state.

Considering the real operation of an interventional surgery, the position and angle of the TACE robot needs to be adjusted by the robot manipulator arm to prevent the guide wire/catheter from warping in real time during the delivery movement because of the long unsupported section of the guide wire/catheter. Therefore, the force-sensing module should work not only in the horizontal working state but also in the inclined working state, as shown in Figure 10.

**Figure 11:**
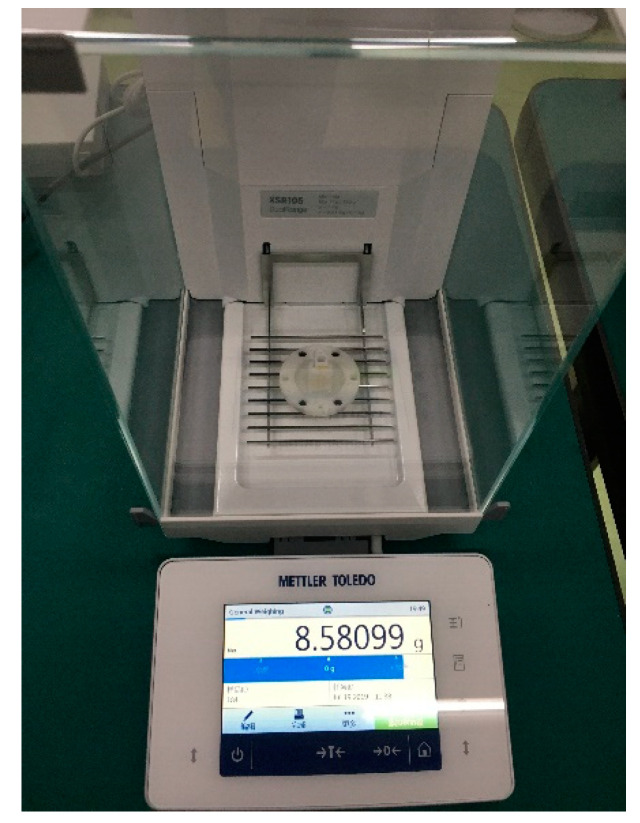
Guide wire fixture weighing.

In the inclined working state, it is necessary to reduce the fixture gravity and friction to reduce the influence of the wire resistance detection, and the weight of the guide wire fixture of the TACE robot is 8.5 g (as shown in Figure 11) while the friction *F_f_* is minimized through contact surface polishing.

**Figure 12:**
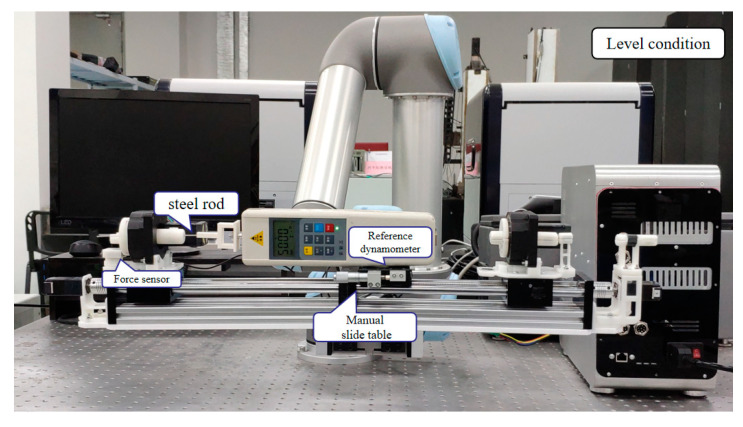
Experimental principle of the detection module.

To evaluate the effect, a steel rod was used to replace the guide wire which was clamped by the guide wire fixture to carry out the time-varying force experiments. In addition, the test value of the dynamometer (HP-5, Yueqing Handpi instruments Co., Ltd., Wenzhou, China) is as the reference. The corresponding time-varying force was measured by the force-sensing module is shown in Figure 12. The matching relationship between the force sensor and the reference dynamometer was tested by adjusting the manual slide table and the steel rod was used to transfer the time-varying force.

**Figure 13:**
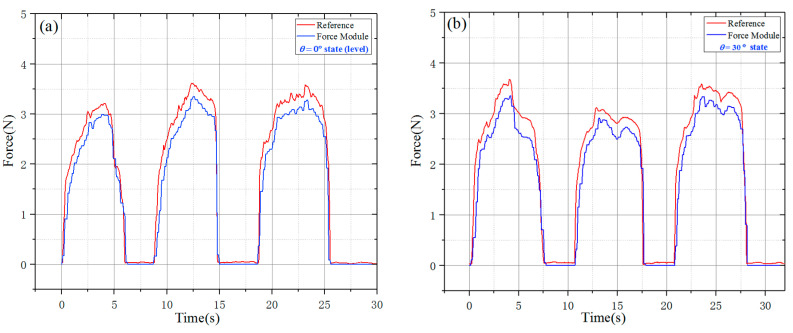
Results of time-varying force experiment (**a**) Horizontal state test result; (**b**) Inclined working state (30°) test result.

The time-varying force experiment was carried out in the horizontal state and the incline state of 30°. The value of the dynamometer could be taken as the *F_G_* in Formula (2). As shown in Figure 13, the force-sensing module could well match the reference dynamometer when the force of the sensor was less than 2.5 N, but the larger force would result in a higher error especially in the incline working state because of the non-negligible friction force, the sliding of the steel rod and the change in axiality. It is believed that the friction force could be effectively reduced and more accuracy results could be achieved by improving the machining quality of the parts of the force-sensing module and adopting mature injection molding process.

Generally, the TACE robot could well measure the resistance of the guide wire with the devised perceptual structure, especially the measurement of the trend of the small force change.

### 3.2. Auxiliary Bending Feedback Method

Placing the sensor in right place of the interventional robot to enrich the sense ability of interventional robot has always been the research highlight. In previous literature [31,32], researchers designed functional guide wire/catheter with fiber optic or set micro sensor on the tip of the guide wire/catheter, and they were successfully tested in the phantom model. However, such studies are hard to replicate in vivo trials because of the difficulty in encapsulation of conducting wire, size reduction, and disinfection.

The force-sensing module described in Section 3.1 could detect the force of the guide wire in the delivery movement well, but the bending of extracorporeal part of the guide wire/catheter would affect the force-sensing result, because the rigidity of the guide wire is gradually reduced from the end to the tip and the catheter is usually soft throughout. This kind of bending is unexpected, but it could be used as a feedback signal to improve the control robustness, therefore, the auxiliary bending feedback method is first proposed by placing the film sensor in extracorporeal part of the guide wire/catheter.

**Figure 14:**
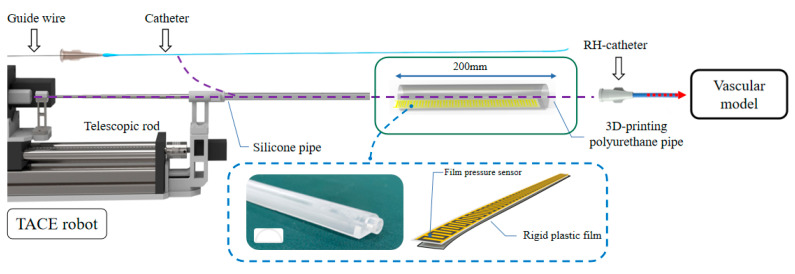
The schematic of auxiliary bending feedback method.

The schematic of auxiliary bending feedback method is shown in Figure 14. The part of the guide wire/catheter in TACE robot could be restrained and supported by the telescopic rods. A 3D-printing polyurethane (PU) pipe is used to support the unrestrained part of the guide wire/catheter between the vascular model and the robot. One end of the PU pipe was connected to the silicone pipe and the other end was connected to the RH catheter. Compared with the silicone pipe, the diameter of the 3D-printing PU pipe is larger. No matter the silicone pipe or PU pipe, it plays a supporting role in the process of catheter delivery, and deformation is easy to be captured in this region once the delivery of the catheter is blocked, and it also plays a role of timely buffering and feedback in case of obstruction.

**Figure 15:**
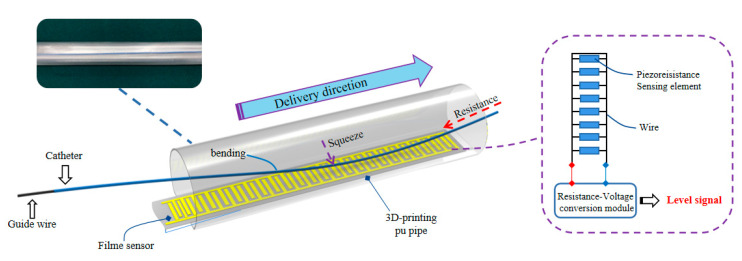
Feedback principle of the film sensor.

A commercial 20 mm long piezoelectric film sensor (RFP620A, YuBo Intelligent technology Co., Ltd., Hangzhou, China) was put into the PU pipe, and the film sensor was fixed with a section of rigid plastic film in the bottom which could reduce interference from its own bending as shown in Figure 15. The actual bending of the guide wire/catheter would be multi-point compression, the film sensor could be regarded as a parallel circuit of multiple variable resistors (the resistance is reduced due to compression), The output voltage of the film sensor could be used as a feedback signal of bending with the relative rules.

**Figure 16:**
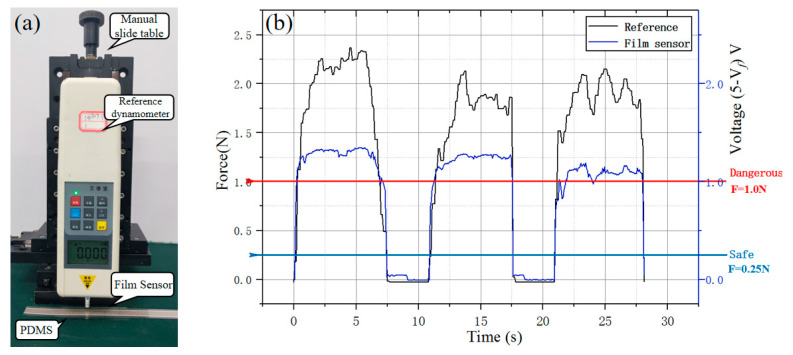
Film sensor verification (**a**) Experiment set-up; (**b**) Film force sensor test mapping curve.

The film sensor based on inverse piezoelectric effect was placed on polydimethylsiloxane (PDMS) for force calibration test with time-varying force by twisting the manual slide table (Figure 16), A resistance-voltage conversion module (RFP-ZHII 5 V, YuBo Intelligent technology, Co., Ltd.) was used to acquire the resistance signal and transform the signal into the amplified analog voltage signal (*V_f_*). From Figure 16b, it is a linear mapping between the transformed analogy voltage and the force when the pressure force is less than 1 N. Meanwhile, combined with subsequent model experiments, the catheter delivery will not be greatly affected by deformation when the value of the film sensor that fixed in the 3D-printing Pu pipe is less than 1 V.

In addition, the guide wire is placed into the target position by the joystick first, and the guide wire could provide guidance and support for the catheter to the target position, obstruction of catheter delivery is relatively rare, its design mainly considers the sudden occurrence of possible vasospasm of TACE. Therefore, the collected pressure signals are simply divided into three levels (safe, middle, dangerous) and used as an auxiliary motion feedback reference such as a threshold switch in the assessment of the possible state of the catheter delivery. In addition, it is also monitored for the whole safety strategy.

### 3.3. Safety Protection Strategy

**Figure 17:**
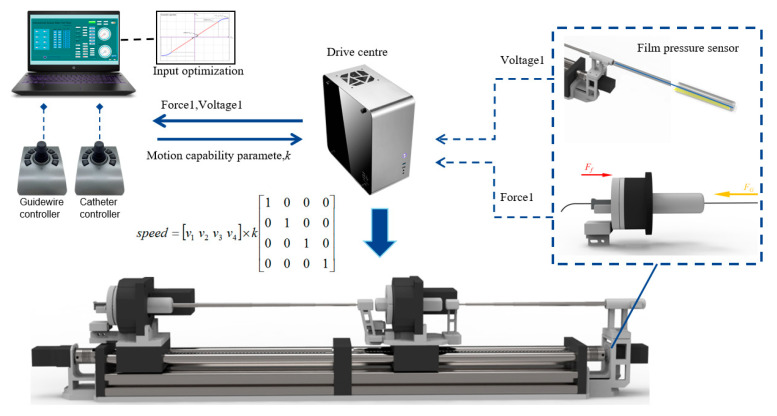
TACE robot design framework.

The schematic of safety protection strategy of TACE robot is shown in Figure 17. In addition, the output of the force-sensing module and the auxiliary bending method are combined in the customized strategy. A rule based on a logic switch is designed to constrain the behavior of the delivery motion of the catheter/guide wire. The voltage of the force sensor is homoplastically divided into three levels to constraint the speed of the TACE robot in the experiment of vascular model. Three assessment levels for the pressure sensor (safe, middle, dangerous) and three assessment levels for the film sensor (safe, middle, dangerous) were used to construct nine evaluations of TACE robot work conditions.

To make the effectiveness of the system more intuitive, nine coefficients of different evaluation conditions were designed based on the numerical value of the force-sensing module and the auxiliary bending feedback voltage of the film sensor, as shown in Table 2. The grade evaluation of the force-sensing mechanism plays a major role in the constraint coefficient, while the grade evaluation of the film sensor plays a secondary role in the determination of the constraint coefficient collectively.

During the operation, the surgeon’s control of the joystick would determine the TACE robot’s 4-DOF motion and speed. The TACE robot would monitor the force signal of the designed force sensor and the analog voltage signal of the film sensor every 10 ms, the motion constraint parameter of the robot is maintained the value of 1 when the conditions of the force sensor and the film sensor are both evaluated safe and once the evaluations change, the motion constraint will be changed less than 1 ms and the speed of the guide wire/catheter could be adjusted automatically according to the motion constraint coefficient. For example, when the film sensor captures a bending feedback larger than 1 V, while the force sensor simultaneously captures a safety feedback less than 0.6 N, the motion constraint coefficient of TACE robot would be 0.3, after algorithm conversion, the speed of the corresponding axis will be changed to 0.3 times of the original output quickly in one communication cycle.

It is also worth mentioning that in order to make the operation more comfortable and achieve better operation stability of the joystick, the algorithm of the robot protection strategy is designed to reset the value of the constraint coefficient only if the hand rocker goes back to original position, or it can only be replaced by a stricter and smaller value of the velocity constraint.

**Figure 18:**
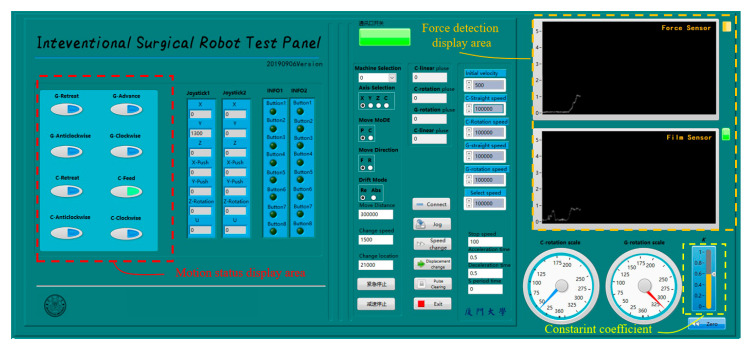
Human–computer interface of the TACE robot.

A human–computer interface was developed by LabView (National Instruments Co., Austin, TX, USA), as shown in Figure 18. The motion state of the TACE robot and the force information was displayed in real time to provide the reference for the surgeons. The color of green, yellow, red in the upper right corner indicates safe, medium risk, and danger, respectively. The constraint coefficient is displayed in the lower right corner.

### 3.4. Experimental Results

**Figure 19:**
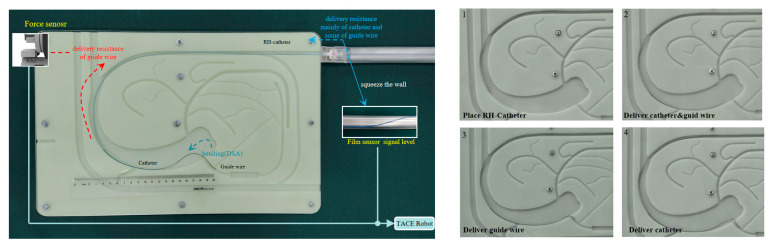
Experiments of vascular model.

A 3D-printing vascular model is designed to simulate the hepatic portal system, as shown in Figure 19. The vascular model was used to test the reliability of the safety protection strategy of the TACE robot. A 0.035-Inch guide wire and a 2.8-French catheter (Cook Medical Co., Bloomington, IN, USA) were employed in the experiment.

The film sensor was fixed on the PU pipe and the guide wire/catheter were placed in the TACE robot. The TACE robot which was held by the robot manipulator arm was adjusted to the same height of the vascular model to ensure PU pipe straight and level to reduce the error of the film sensor, as shown in Figure 19. Meanwhile, the guide wire/catheter and the RH catheter were lubricated with appropriate water before the test.

**Figure 20:**
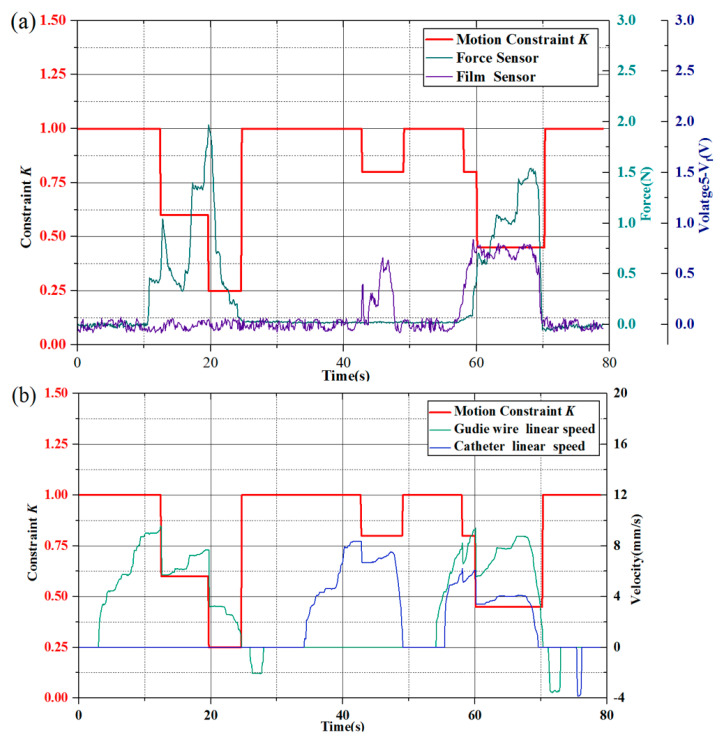
Vascular model experiment result (**a**) Relationship between sensors and constraint coefficient; (**b**) Actual linear motions constraint effect of the guide wire/catheter.

The transformed output speed of the joystick, the force from the force sensor and the voltage from film sensor were collected in the computer background as show in Figure 20.

Independent delivery and rotary movements, coupled delivery and rotary movements of the guide wire/catheter were tested in the experiment of the vascular model. The guide wire/catheter were delivered into the branch successfully by the operation of remote-controlled joysticks. The safety protection strategy based on two sensors was also validated in the vascular model experiments.

The output speed of the joystick, the force of the force sensor and the voltage of film sensor were drawn in Figure 20. In the model test experiment, it could be seen that 0–30 s: only the guide wire was delivered. During this period, the guide wire could still be sent under the resistance less than 2 N that detected by the force-sensing module, while the bending deformation influence the catheter in PU pipe is very small.

About 35–50 s: only the catheter was delivered. The catheter was delivered forward and had a certain extent in the PU pipe induction section, the friction would give the guide wire a tendency to move forward rather than squeezes the force sensor and the recoil of the guide wire caused by catheter deformation is too weak, so the force sensor value remained the same.

In TACE robot system, force-sensing module could measure the resistance of the guide wire delivery and auxiliary bending feedback method is mainly to evaluate the level of catheter delivery, their work is relatively independent.

About 55–70 s: simultaneously delivery of the guide wire/catheter by joysticks. At this period, the film sensor and the force sensor play a role in determining the motion constraint coefficient *k* in the feedback of the guide wire and the catheter, respectively. When they worked together, the motion constraint coefficient often would be lower in the test.

In allusion to safety protection strategy, as shown in Figure 20a, the guide wire encountered the resistance at approximately 10–20 s, the detected force increased immediately and drastically. The protection strategy took effect and the delivery speed of the guide wire was restricted in time, the constraint parameter of the delivery speed reduced to 0.6 and 0.25 in turn and the detected resistance was also reduced. At approximately 60–70 s, the guide wire and catheter were operated to advance simultaneously, the force sensor and the film sensor worked together at this stage. In addition, the film sensor mainly works on evaluating the working levels of the catheter by the bending, the constraint parameter of the robot system reduced to 0.45 under the corresponding test condition. In the above process, the two sensor signals were effectively fed back to the TACE robot and the speed of the TACE robot was constrained in time which could avoid potential aggressive delivery.

## 4. Conclusions

In this paper, a 4-degree-of-freedom remote-controlled robotic system is proposed for transcatheter arterial chemoembolization and the TACE robot could realize the maximum linear delivery distance of 200 mm and unrestricted angular rotation of the guide wire/catheter. The master–slave robot system was established and the independent control and coupling control could be realized by the remote-control system. Two operation mode were proposed, and the customized sigmoid function was used to stable and smooth the speed of the TACE robot in speed control mode. The force-sensing module was designed to detect the resistance in the delivery movement of the guide wire/catheter. The force-sensing module is light weight and could detect the resistance well under the resistance of 2.5 N. The safety protection strategy was proposed based on bending auxiliary feedback method and the force-sensing module. The motion constraint coefficient could realize the speed constraint of the robotic system. Through the results of the vascular model experiment, the effectiveness of the safety protection strategy by combining the feedback of the force sensor and the film sensor was verified.

In future, the injection molding process would be adopted in the robotic system, the fixtures of guide wire and catheter would be designed as disposable magnetic adsorption structure to improve the efficiency of the operation and reduce the volume and weight, and new kinds of film sensors would be adopted to improve the effect of continuous velocity constraint instead of the existing step constraint framework.

## Figures and Tables

**Table 1:** The movement parameters of the TACE robot.

Object	Linear Speed	Rotary Speed	Range of Linear Motion	Range of Rotary Motion
Guide wire	0–12 mm/s	0–1200°/s	0–200 mm	No limitation
Catheter	0–12 mm/s	0–1200°/s	0–200 mm	No limitation

**Table 2:** The motion constraint coefficient *k* of TACE robot.

Condition		Force Sensor		
		Safe (<0.6 N)	Middle	Dangerous (>2.1 N)
**Film Sensor**	Safe (<0.25 V)	1	0.6	0.25
	Middle	0.8	0.45	0.1
	Dangerous (>1 V)	0.3	0.2	0
